# Tailored implementation of national recommendations on fall prevention among older adults in municipalities in Norway (FALLPREVENT trial): a study protocol for a cluster-randomised trial

**DOI:** 10.1186/s13012-024-01334-2

**Published:** 2024-01-25

**Authors:** Maria Bjerk, Signe A. Flottorp, Are Hugo Pripp, Henning Øien, Tonya Moen Hansen, Robbie Foy, Jacqueline Close, Siv Linnerud, Therese Brovold, Rune Solli, Nina Rydland Olsen, Dawn A. Skelton, Elisabeth Rydwik, Jorunn L. Helbostad, Gro Idland, Linda Kvæl, Edgar Vieira, Kristin Taraldsen

**Affiliations:** 1 Department of Rehabilitation Science and Health Technology, Faculty of Health Sciences, Oslo Metropolitan University (OsloMet), Oslo, Norway; 2 Division for Health Services, Norwegian Institute of Public Health, Oslo, Norway; 3 Department of General Practice, Institute of Health and Society, University of Oslo, Oslo, Norway; 4 Oslo Centre of Biostatistics and Epidemiology, Oslo University Hospital, Oslo, Norway; 5 University of Leeds, Leeds, UK; 6 Neuroscience Research Australia, Randwick, NSW Australia; 7 Prince of Wales Hospital, SESLHD, Randwick, NSW Australia; 8 Department of Health and Functioning, Faculty of Health and Social Sciences, Western Norway University of Applied Sciences, Bergen, Norway; 9 Research Centre for Health (ReaCH), Department of Physiotherapy and Paramedicine, School of Health & Life Sciences, Glasgow Caledonian University, Glasgow, UK; 10 Women’s Health and Allied Health Professionals Theme, Medical Unit Occupational Therapy and Physiotherapy, Karolinska University Hospital, Solna, Sweden; 11 Department of Neuromedicine and Movement Science, Faculty of Medicine and Health, NTNU, Trondheim, Norway; 12 Agency for Health, Municipality of Oslo, Oslo, Norway; 13 Department of Housing and Ageing Research, Norwegian Social Research – NOVA, Oslo Metropolitan University (OsloMet), Oslo, Norway; 14 Department of Physical Therapy, Florida International University, Miami, FL USA

**Keywords:** 3–10 Implementation science, Implementation strategies, Cluster-randomised trial, Older adults, Fall prevention, Municipal health services, Implementation intervention, National recommendations, Guidelines

## Abstract

**Background:**

Despite substantial research evidence indicating the effectiveness of a range of interventions to prevent falls, uptake into routine clinical practice has been limited by several implementation challenges. The complexity of fall prevention in municipality health care underlines the importance of flexible implementation strategies tailored both to general determinants of fall prevention and to local contexts. This cluster-randomised trial (RCT) investigates the effectiveness of a tailored intervention to implement national recommendations on fall prevention among older home-dwelling adults compared to usual practice on adherence to the recommendations in health professionals.

**Methods:**

Twenty-five municipalities from four regions in Norway will be randomised to intervention or control arms. Each municipality cluster will recruit up to 30 health professionals to participate in the study as responders. The tailored implementation intervention comprises four components: (1) identifying local structures for implementation, (2) establishing a resource team from different professions and levels, (3) promoting knowledge on implementation and fall prevention and (4) supporting the implementation process. Each of these components includes several implementation activities. The Consolidated Framework for Implementation Research (CFIR) will be used to categorise determinants of the implementation process and the Expert Recommendations for Implementing Change (ERIC) will guide the matching of barriers to implementation strategies. The primary outcome measure for the study will be health professionals’ adherence to the national recommendations on fall prevention measured by a questionnaire. Secondary outcomes include injurious falls, the feasibility of the intervention, the experiences of the implementation process and intervention costs. Measurements will be carried out at baseline in August 2023, post-intervention in May 2024 and at a follow-up in November 2024.

**Discussion:**

This study will provide evidence on the effectiveness, intervention costs and underlying processes of change of tailored implementation of evidence-based fall prevention recommendations.

**Trial registration:**

The trial is registered in the Open Science Registry: 10.17605/OSF.IO/JQ9T5. Registered: March 03, 2023.

**Supplementary Information:**

The online version contains supplementary material available at 10.1186/s13012-024-01334-2.

Contribution to the literature

- The evidence on fall prevention is substantial, and evidence-based global guidelines on fall prevention have recently been published. However, there is a lack of research on how to locally tailor and implement these comprehensive guidelines into clinical practice in municipality settings.
- The FALLPREVENT study has a robust design based on previous studies including a co-creation process and a feasibility study. These methodological contributions can advance the field of research in real-world settings and offer guidance to future studies.
- This cluster-randomised trial conducted within 25 city districts/municipalities in Norway will provide knowledge on the effectiveness of an implementation intervention to enhance adherence to national guidelines on fall prevention in a municipality setting and will be important for future policy and practice.

## Background

Globally, falls and fall-related injuries are major contributors to disability and death in older adults aged 65 years and older, and a significant public health concern [1]. Norway has among the highest reported hip fracture incidence rates in the world, and the highest incidence of other fall-related injuries requiring health care in Western Europe [2, 3]. Reducing falls among older adults is an international health priority [1, 4]. Multiple systematic reviews and meta-analyses provide evidence for the effectiveness of fall prevention programmes [5–17]. A recent global initiative published new global guidelines for fall prevention and management for older adults and highlighted the importance of flexible implementation strategies tailored to local contexts and resources [4].

Despite abundant research on fall prevention, the implementation of evidence-based fall prevention into practice has been slow and limited [18–21]. We are not aware of previous studies on the implementation of fall prevention using tailored strategies to address identified barriers or facilitators, or determinants of practice. A Cochrane review concluded that tailored interventions addressing determinants of practice can be effective, but the effect is variable and tends to be small to moderate [22]. None of the 32 studies included in this review targeted fall prevention. Determinants of practice can be grouped into seven domains: the guideline, the individual health professionals, the health care system, patients, professional interactions, incentives and resources, capacity for organisational change, and social, political and legal factors [23].

Implementation challenges can be related to health care professionals, the health care system, older adults themselves and their families [19]. For instance, health care professionals often fail to refer older adults to fall prevention interventions after a fall injury [24], and few community dwellers at risk of falls recognise their own risk and prioritise preventive interventions [25]. For example, exercise, which is the single most effective fall prevention strategy, has shown uptake rates in communities as low as 10% [5, 8, 26, 27]. Limited implementation of fall prevention programmes by health professionals could be explained by barriers, including lack of knowledge and skills, time and financial constraints, and the complexity of health and social care environments [28]. Moreover, older adults may be reluctant to report falls, and their underestimation of their own fall risk, fear of falling, and stigma related to falls might limit their participation in fall prevention programmes [29]. Nevertheless, The Falls Management Exercise (FaME) trial is one example of a fall prevention exercise programme delivered in a “real-world” setting that was implemented with high fidelity among older adults, albeit with some loss of programme fidelity [30].

The organisational structure of the health care service also plays a central role in implementation, with success likely to depend on the service’s perceived need for innovation, sufficient capacity for change, decision-making authority and leadership [31]. In Norway, over the last decade, more tasks have been transferred from specialist care to the municipalities, including providing appropriate and coordinated care for older adults [32]. The range and growing complexity of tasks now included in municipality care put greater demands on both municipal capacity and expertise [33]. In Norway, the National Directorate of Health is responsible for developing national clinical recommendations. Currently, new national recommendations on fall prevention among older adults are being developed. However, publishing national recommendations on fall prevention is unlikely to be sufficient by itself to bring about a major change in clinical practice given the challenges around the complexity, relevance and usability of the recommendations [34].

Most implementation studies to date have evaluated the effectiveness of implementing fall prevention exercise interventions and explored different elements of the implementation of fall prevention services. However, to our knowledge, none has evaluated the implementation of national recommendations and broader fall prevention interventions tailored to determinants of fall prevention in general and to the local municipal health care services more specifically. Thus, our research questions are the following: [1] What are the effects of a tailored implementation intervention compared to usual practice on health professionals’ degree of adherence to national recommendations for fall prevention?; [2] What are the effects of the implementation intervention on feasibility, the implementation process from the perspective of individuals involved in implementation activity, resource use and injurious falls rate?; [3] What are the economic costs of the implementation intervention? and [4] How will health professionals and managers experience participating in the implementation programme? We hypothesise that the tailored implementation intervention will increase the degree of adherence to the national fall prevention recommendations among health professionals in the municipalities and city districts. Furthermore, increased adherence will reduce the frequency of falls resulting in injuries requiring the attention of health care services.

## Methods

### Trial design

**Fig. 1:**
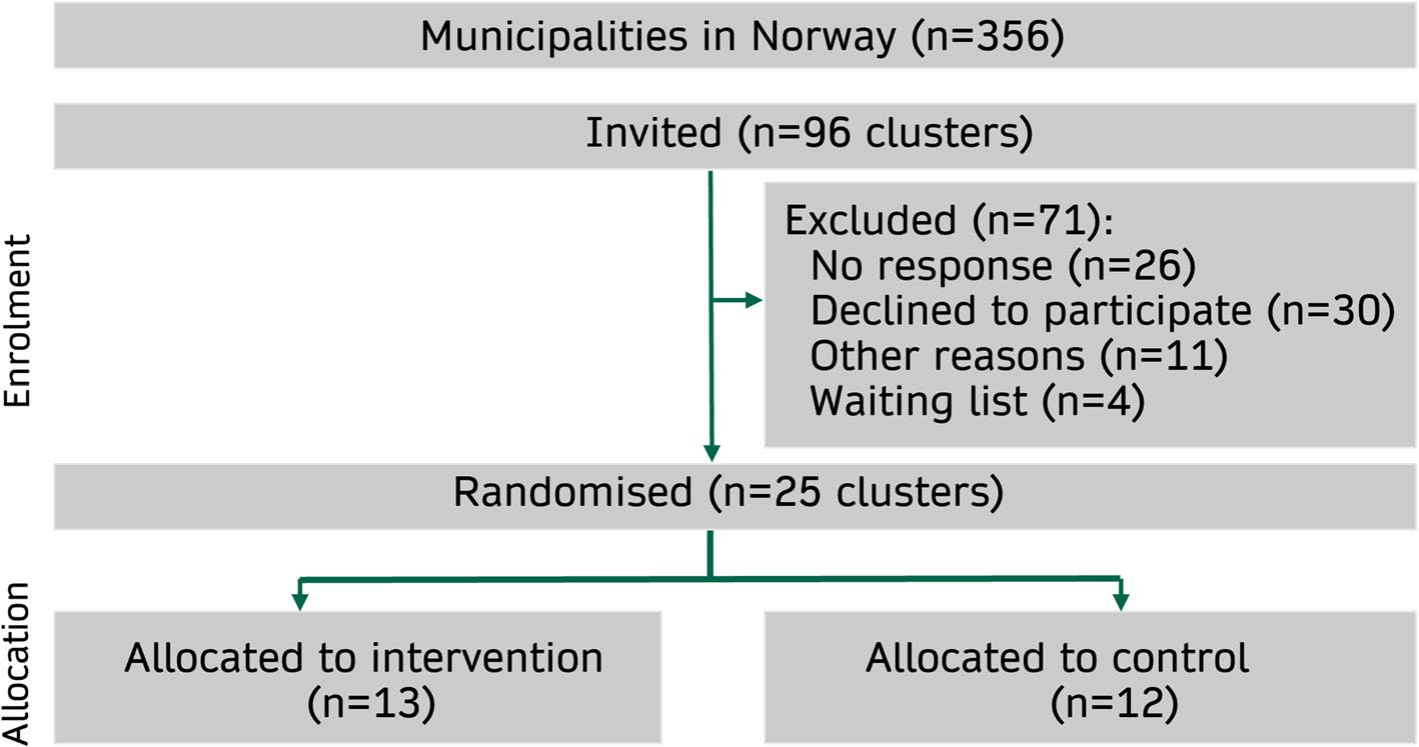
Flow chart

The study is a cluster randomised trial with randomisation at the municipality level and 1:1 allocation to parallel groups. A sample of 25 municipalities and city districts will be randomised to either an intervention group receiving the implementation intervention, including a 4-month planning phase and a 4-month action phase or a control group continuing practice as usual. Recruitment of municipalities and city districts started in April 2023 and was completed in July 2023 (see Fig. 1). The start-up was September 1st, 2023, with an 8-month intervention period and a 14-month follow-up in November 2024. Our reporting in this protocol adheres to the SPIRIT checklist [35] and CONSORT checklist for cluster RCTs [36].

### Study setting and population

The setting for this trial is Norwegian municipalities and city districts in Norway: the east, middle, west and southeast region. Municipalities are eligible if they have the potential to recruit approximately 30 health professionals as responders and have voluntarily signed a collaboration agreement. In larger municipalities, city districts are eligible if they are defined as geographical areas within a city with their own decentralised public administration responsible for health services including care for older adults.

After the inclusion of municipalities and city districts, a sample of 30 health professionals primarily working with older adults (65 +) will be recruited within each cluster. Eligible health professionals are nurses and assistants in home health care services, general practitioners, physiotherapists, occupational therapists and managers working at different levels within the municipality/city district. Each cluster must recruit participants from at least three different professions. One identified person (manager or health professional) from each cluster will function as a coordinator, responsible for collecting the data at the municipality level and being the contact between health care professionals participating in the intervention and the researchers in FALLPREVENT. Data at the patient level (for example falls and fall injuries) will be collected using the municipalities’ routinely collected health data.

### Data collection and randomisation

**Fig. 2:**
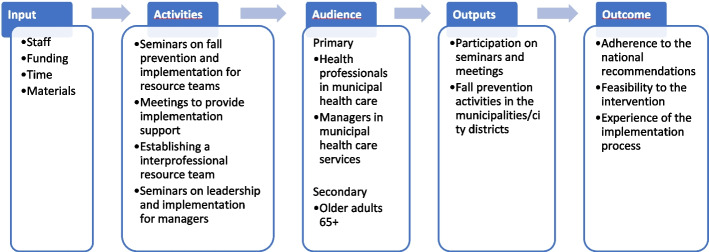
Logic model for the FALLPREVENT intervention

We will collect data at baseline (T0: August 2023) prior to randomisation, immediately after the end of the intervention period (T1: May 2024) and then 6 months later (T2: November 2024). Data for the study will be drawn from multiple sources. We will send three online questionnaires, including an informed consent form, to the 30 health professionals recruited from each cluster at T0, T1 and T2. The coordinators will collect the data at cluster level and report this by use of an online questionnaire at T0, T1 and T2. We will collect data on resource use for the intervention group during the intervention period, and we will draw data on injurious falls retrospectively from health registers. We will collect qualitative data from interviews with health professionals and managers in the intervention group at T1 and T2. Figure 2 presents a logic model for the FALLPREVENT intervention, summarising the proposed links between the input, activities, audience, output and outcomes.

Randomisation is undertaken following the completion of T0, using a computer program (RALLOC Stata module), which will generate a sequence of treatments randomly permuted in blocks. The clusters are allocated consecutively to this sequence and included in the trial. The project statistician is responsible for the randomisation process and forwards the result to the research team who then informs the coordinators.

Pre-intervention measures at T0 are collected before randomisation. At T1 and T2, blinding of the health professionals is not possible since they participate in the intervention workshops and are thus aware if they are included in the control group or intervention group. Also, blinding of the researchers is not possible since they are responsible for distributing the questionnaires and conducting the seminars in the intervention. The statistician performing the statistical analyses will be kept blind to intervention allocation.

### Intervention

Our implementation intervention will target the national recommendations for fall prevention among older adults, developed and published by the Norwegian Directorate of Health. These recommendations have been developed in a process lasting approximately one year, comprising relevant systematic reviews and inputs from a reference group including clinicians, researchers, user organisations and managers. A draft of these recommendations has been made available through an official hearing in July 2023, and the final version will be published in November 2023 [37]. The national recommendations are based on the World guidelines for fall prevention and management for older adults [4], but this trial focuses solely on the recommendations for municipal health care as home-dwelling older adults at 65 + is our population of interest.

We developed the FALLPREVENT implementation intervention through several steps, with the overall aim that the municipalities and city districts will be enabled to translate the national recommendations into their own clinical context and to tailor the interventions to determinants of practice (barriers and facilitators), both in general, but also at the local level. Thus, the implementation strategy allows for local tailoring to suit the complexities of fall prevention and the municipal health care context [22]. Previous research has highlighted the importance of addressing determinants and selecting suitable strategies at different contextual levels in order to implement fall prevention interventions in a community setting [38, 39]. We thus conducted a co-creation process involving researchers, users of the health care service and health professionals identifying relevant barriers and facilitators in the development phase [40]. In line with the results, we developed the first version of the implementation intervention. Intervention components included manager commitment, establishing a resource team from different professions and levels, promotion of knowledge about fall prevention and implementation, and support in the implementation process. We also included suggestions from previous research on implementation strategies in different settings within municipality care, such as active learning arenas, including workshops and tutorials, tools to structure the process and support from the management [41, 42].

The first version of the implementation intervention was tested in a feasibility study in two city districts in Oslo from January to April 2023. The 12-week intervention period consisted of a planning phase and an intervention phase with one seminar for the managers and four seminars for an interprofessional resource team from each city district. To categorise local determinants, we used the Consolidated Framework for Implementation Research (CFIR), followed by the CFIR-ERIC to connect the determinants with relevant implementation strategies [43, 44]. We have used the results from this feasibility study to refine our implementation intervention, by including other relevant strategies and activities (see Supplementary Table S1) and by developing the questions for the outcome measure on adherence to the national recommendations.

The FALLPREVENT implementation intervention consists of two phases, a planning phase where the clusters will identify a structure for implementation and develop an implementation plan based on local challenges and needs, and second, and an action phase where the planned activities will be conducted, and sustainment ensured.

**Fig. 3:**
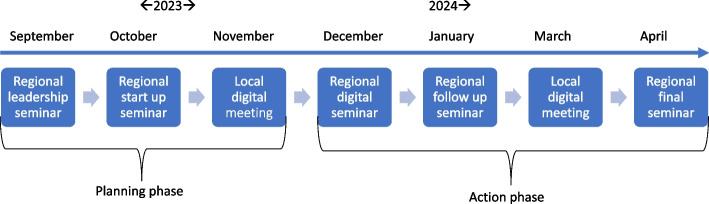
Timeline of the implementation intervention

The key components of the implementation intervention are as follows: (1) Identifying local structures for implementation, (2) establishing a resource team from different professions and levels, (3) promoting knowledge on implementation and fall prevention among home-dwelling older adults and (4) supporting the implementation process. These components include several more specific implementation activities, which we describe in the following paragraphs. Figure 3 shows a timeline of the implementation intervention.

#### Identifying local structures

To start, local structures for the implementation intervention will be identified and established. Examples of local structures can be departments or teams relevant to fall prevention. Identifying these structures in each municipality or city district is of importance due to the heterogeneity of Norwegian municipalities, in terms of geography, demography, prioritisation and organisation of municipal healthcare. A structure for implementation should determine resource use and define who is responsible for the implementation: the planning, the implementation in practice and how local follow-up should be carried out. Managers play a central role in identifying and establishing the local structures and their commitment is essential. To promote manager commitment from the start, we will conduct a regional seminar including five to six managers from each municipality or city district, with the title “Leadership in implementation in municipal health care services”. The participating managers can be situated at different levels in the health service, depending on the local organisation.

#### Establishing a resource team

The manager will also be responsible for organising a resource team consisting of 4–6 health professionals, including a manager. The resource team should be diverse and represent different health professions to be able to influence different sectors within the multi-professional health care services in the municipalities. The manager included in the resource team can be either an informal manager, for instance, a health professional with a team leader role, or a formal manager, with personnel responsibility. This team will lead the development of the implementation plan and the implementation process in their municipality/city district, supported by the researchers in FALLPREVENT through seminars and meetings.

#### Promoting knowledge on implementation and fall prevention

There will be four 1-day seminars for the resource group, where one is digital. These will be arranged within the regions. The main aim of the seminars is to provide the resource teams with knowledge and materials on fall prevention and implementation. An overview of the seminars and main aims are shown in Table 1.

**Table 1:** Overview of the seminars and main aims of the seminars

Seminars	Main aim
Leadership seminar	Provide knowledge on fall prevention and implementation and further the manager’s role in implementing evidence-based fall prevention
Start-up seminar	Provide knowledge on fall prevention and implementation Provide knowledge on evaluating current practice, adapting evidence-based knowledge on fall prevention to local context, and conducting a stakeholder analysis
Follow-up seminar digital	Provide knowledge on determinants (barriers and facilitators) for implementation of national recommendations on fall prevention, goal setting, and ways of monitoring changes
Follow-up seminar	Provide knowledge on linking determinants and implementation strategies, and further implementation activities
Final seminar—evaluation	Share knowledge and experiences of conducting the implementation intervention Provide knowledge on the sustainability of interventions

At the start-up seminar and at the digital follow-up seminar, the resource teams will mainly work on an implementation plan, which is an important tool throughout the intervention period (see Supplementary Table S2). The implementation plan will be based on the Norwegian adapted version of the implementation toolkit, *Implementation of Best Practice Guidelines* [45], based on the action cycle of the Knowledge to Action framework [46]. Additionally, as part of the implementation plan, we will also use the CFIR [43, 47] to further elaborate and categorise determinants of the implementation process and match the barriers to the ERIC compilation of implementation strategies [44].

Next, the implementation strategies and activities described in the local implementation plans will be carried out in practice. In this phase, we will arrange two seminars, one follow-up seminar where the focus will be on the implementation activities, and one final seminar where the aim is to share experiences and present results from the implementation process and further make plans for sustainability.

#### Supporting the implementation process

Beyond the support already described above, the municipalities and city districts will be supported between the seminars. This support consists of access to materials, local meetings led by members of the FALLPREVENT group and homework between meetings. A toolkit of materials for implementing fall prevention interventions will be available to the intervention clusters at the second seminar. This toolkit includes, among other materials, posters, PowerPoint presentations, brochures on fall prevention directed to older adults, examples of clinical cases and quizes. Two 1-hour digital meetings will be scheduled for each of the intervention clusters, to provide specific support based on local needs. Furthermore, the FALLPREVENT group will provide support to intervention clusters, if needed, during the intervention phase of the project.

### Control group

The municipalities and city districts in the control group will have access to the national recommendations on fall prevention, but they will not receive any implementation intervention or support. After completion of the research project, the municipalities and city districts in the control group will be invited to a seminar where we will give presentations including results from the trial. Additionally, control group municipalities and city districts will then be given access to the material on fall prevention and on implementation developed through the FALLPREVENT project.

### Primary outcome

The primary outcome is the degree of adherence to the national recommendations on fall prevention as reported by health professionals. As no relevant existing questionnaires were available, we developed a custom-made questionnaire, guided by a similar trial [48]. Each item in our questionnaire is related to a clinical practice recommendation, giving a total of ten questions. Health professionals will grade their answers according to a five-point Likert scale, providing sub-scores (0–5) and a total sum score of up to 50 points. Face validity of our questionnaire was assessed through a think-aloud discussion with health professionals in a feasibility trial, and several clarifications were made to the final questionnaire (Linnerud et al.: The feasibility of an implementation strategy for preventing falls in home health services, unpublished).

### Secondary outcomes

#### Demographics and other data at cluster level

At the municipality/city district level, we will include data on the total number of home-dwelling older adults (65 years and older) and the total number of older adults receiving the following services: safety alarm service, home nursing, practical assistance, rehabilitation services, preventive services and fall prevention interventions. Additionally, we will collect data on other ongoing interventions on fall prevention, such as exercise groups for older adults with a low risk of falls, exercise groups for older adults with a medium risk of falls, assessment and interventions for older adults with a high risk of falls and systems for monitoring falls. We will also collect data on the distribution of information and education to service users and health care professionals, in addition to if and how fall prevention is included in strategic documents. If available, data on the number of falls in older adults will be collected.

#### Fall injuries

The number of fall injuries per cluster will be collected along with registered diagnosis and procedure codes capturing sprains and fractures in the Municipal Care Registry (KPR) and Norwegian patient registry (NPR).

#### Feasibility of the clinical recommendations

At an individual level, health professionals within each cluster will answer questions related to the feasibility of the clinical recommendations by use of the Feasibility of Intervention Measure (FIM), a four-item questionnaire with a five-point scale ranging from completely disagree to completely agree [49].

#### Experience of the implementation processes

We will use the Normalization Measure Development Questionnaire (NoMAD) to measure the implementation processes from the perspective of health professionals [50, 51]. This questionnaire is suited when evaluating the implementation of complex interventions in health care [52]. It is a questionnaire consisting of 20 items with a five-point scale ranging from completely agree to completely disagree.

#### Intervention costs

We will carry out an economic evaluation of the costs of the implementation intervention. During the intervention phase, we will collect data on the costs of the FALLPREVENT intervention. The data will consist of the time-use for the intervention (resource teams, seminars and meetings) and the number of health professionals participating in the meetings and seminars and their job positions and education.

### Interview with health professionals and managers

We will collect qualitative data to explore the participants’ experience of the implementation intervention. We will carry out face-to-face focus group interviews (one to two per region) with six to eight health professionals from the resource groups immediately after the end of the intervention. Topics to be discussed will include their experiences with the implementation intervention, working in an interprofessional team and applying the knowledge on implementation and fall prevention to their clinical practise. In addition, we will perform digital semi-structured interviews with ten to twelve managers from the intervention clusters to discuss their role as managers during implementation and their experiences with the implementation intervention.

### Sample size

We did a sample size calculation based on the primary outcome; health professionals’ adherence to the national recommendations, a ten-item questionnaire with answers scored according to a five-point Likert scale. The sample size calculation accounts for the intra-cluster correlation coefficient, the number of clusters, the number of responders in each cluster and the expected effect expressed as the mean difference between the intervention and the control group in relation to the standard deviation of the outcome within each group [53]. The expected effect is equal to Cohen’s *d* effect size of 0.5 (i.e., the mean difference between groups equal to 0.5 standard deviation of the outcome variable). With an assumed intra-cluster correlation of 0.1 and 12 clusters in the intervention and 12 in the control arm, we require 10 participants in each cluster to obtain 80% statistical power at a 5% significance level—totalling 120 participants in each randomised group. However, to allow for missing data, dropouts and uncertainties about the expected effect and intra-cluster correlation, we will include an average of 30 responders from each cluster.

### Statistical analysis and data management

We will explore the effectiveness of the tailored interventions to implement the national fall prevention recommendations in the municipalities. A statistician blinded to group allocation will undertake the statistical analysis. A complete data analysis plan was finalised and published on April 21, 2023, in the open science framework https://doi.org/10.17605/OSF.IO/EPG72.

The effectiveness of the intervention with the mean (95% CI) or median (IQR) adherence, will be calculated from data recorded by the adherence questionnaire. We will evaluate the change in adherence sum score from baseline to follow-up, for the intervention group compared to the control group. The main intention to treat analyses will include all the 30 responders from each randomised cluster, regardless of protocol fidelity. To provide further insight into the effectiveness of the intervention, per-protocol analyses will be carried out where we will include all participants in randomised municipalities and city districts meeting the study eligibility criteria and with no major protocol deviations affecting the treatment efficacy. To consider cluster effect and repeated measurements, generalised linear mixed model with cluster and subject-specific random intercept will be used as the main method as outlined by Twisk [53]. If the pre-specified statistical model does not converge, we will assess equivalent statistical models with a simpler structure or use generalised estimating equations or robust standard errors. Fixed effects in the model are the outcome variable at baseline as a covariate and time, intervention group and the interaction between time and intervention. It will be compared to basic methods that do not consider cluster effect, e.g., independent sample *t* test, Mann–Whitney *U* tests or chi-square tests. Missing data will be left missing; no imputation methods will be used in the primary statistical analysis.

To assess the economic costs of the intervention we will present a detailed documentation of the incurred costs. The documentation will discern which components, such as costs related to travel time and personnel commitment, are driving the cost of the intervention. To evaluate the cost of the intervention on adherence, we will compute the cost per percentage point increase in adherence sum score. We will conduct a sensitivity analysis to account for uncertainty in cost and effect estimates.

We will design questionnaires in “Nettskjema”, which is a secure data capture tool developed by the University of Oslo, that offers a range of functions to collect, store and analyse data from the desired target group. The researcher will send a link by email to the participants and then the answers are returned to the same portal. The participants will have to provide informed consent before filling out the questionnaire. Questionnaires will be stored and de-identified in OneDrive with access control and login during analysis. After the end of the study, data will be anonymised according to the approval of The Norwegian Agency for Shared Services in Education and Research (SIKT) [54]. Personally identifiable information will be removed or rewritten.

We will record all individual and focus group interviews using a digital voice recorder and transcribe them before data analysis. We will analyse the transcript using reflexive thematic analysis by Braun and Clark [49], using the six-step method to identify, analyse and report qualitative data patterns. Audio recordings will be directly uploaded to and saved in “Nettskjema”. When conducting interviews digitally through Zoom, we will store the recordings safely in Microsoft OneDrive with access control and login.

### Trial status

The trial commenced recruitment in April 2023. In June 2023, a total of 25 clusters (19 municipalities and 6 city districts) were recruited. In July, all 25 clusters had signed and returned the study agreements and were included in the trial. Data collection prior to randomisation started on August 7 and ended on August 31, 2023.

### Dissemination plans

Results will be published in peer-reviewed and scientific journals. We will present the results at national and international conferences and use it in education within health care services. Results will be disseminated regardless of the magnitude or direction of the effects.

## Discussion

This study will evaluate the effectiveness of a tailored implementation intervention to implement national recommendations on fall prevention among home-dwelling older adults in a municipal health care setting. In the intervention group, the fall prevention intervention will be locally tailored to each municipality and city district according to their needs and resources. The local tailoring and modifications to local settings are in accordance with international guidelines for fall prevention for older adults [4]. Moreover, matching departmental programs, such as national recommendations, and research has been recommended to ensure efficient resource allocation [55, 56]. To our knowledge, this is the first study in Norway evaluating health care services´ adherence to newly published national recommendations. It will provide valuable knowledge on implementing national recommendations within a complex municipality setting, and this knowledge can be further transferred to implementing recommendations within other areas of health care.

Fall prevention has been a part of clinical practice in the municipal health care in Norway for many years but is variably delivered. By including a broad spectrum of municipalities and city districts with different geography, demographics and priorities, we anticipate that we will gain knowledge on how to and to what extent fall prevention is delivered in different municipalities and city districts. By introducing a tailored implementation intervention with structure and practical guidance, we may help health professionals within the municipalities to increase their adherence to the national recommendations on fall prevention. We will also gain knowledge on the health professionals’ and managers’ experience of implementing evidence-based fall prevention in their municipalities and city districts and how municipalities can work to implement national recommendations. The study will provide knowledge which can inform the future development of municipal health care services for older adults at low, medium and high risk of falls.

The significant strengths of this study are the anchoring of the implementation intervention in implementation theory and evidence from systematic reviews on implementation interventions, the local tailoring and involvement of stakeholders through co-creation, and the demonstration of its feasibility. We will be able to provide knowledge from diverse clinical practices in Norway by including 25 municipalities and city districts in different regions, with the exception for the northern region. Given the potential contamination between intervention and control clusters, we will instruct the managers in the intervention municipalities to limit their sharing of documents and acquired knowledge.

We will report adherence to the national recommendations by use of self-reporting within a sample of health professionals representing their municipality. Notable considerations are that this questionnaire is self-reported and that it was developed and adapted within the project. The randomised design can help create a fair comparison across the intervention and control conditions. The lack of blinding might introduce a risk of bias. The participants in the intervention condition might be more motivated to report higher adherence than those in the control condition. To provide more data, and to be able to compare a potential impact on clinical outcomes, we will also examine injurious falls from health registers as secondary outcomes. Preferably, we would have included the fall rate in older adults as a measure; however, there are no data routinely collected on falls in the municipalities.

## Conclusion

This study will examine whether and how a comprehensive, tailored intervention, targeting municipal managers and health professionals, increases the implementation of national fall prevention recommendations in practice. It will also inform local strategies and implementation plans tailored to individual resources and teams in municipalities in Norway. Findings from this study will provide policy makers with knowledge on how national recommendations are implemented in the municipalities. Ultimately, we hope that it will substantially contribute to future reductions in falls amongst older adults.

### Supplementary Information

**Additional file 1: Supplementary Table S1.** Implementation strategies, elements defined by stakeholders and activities.

**Additional file 2: Table S2. **Developing a local implementation plan.

## Data Availability

Economic and formative data collection materials are provided as additional files. Complete details on the operationalization of measures using electronic health data are outlined in the Statistical Analysis Plan (available from authors). The trial’s Data Safety and Monitoring Plan and protocol amendments are available from the first author.
