# Supplementary material for: Tailored implementation of national recommendations on fall prevention among older adults in municipalities in Norway (FALLPREVENT trial): a study protocol for a cluster-randomised trial
Source: Implement Sci. 2024 Jan 25;19:5. doi: 10.1186/s13012-024-01334-2 (PMC10811923; doi:10.1186/s13012-024-01334-2)
Supplement: Supplementary file 1 — Additional file 1: Supplementary Table S1. Implementation strategies, elements defined by stakeholders and activities. [file 13012_2024_1334_MOESM1_ESM.docx]

**Supplementary table S1.** Implementation strategies, elements defined by stakeholders and activities.

| **Relevant implementation strategies (CFIR-ERIC)** | **Implementation elements defined by stakeholders (co-creation process)** | **Implementation activities in the intervention** |
| --- | --- | --- |
| Adapt and tailor to context | Knowledge on fall prevention and implementation    Support in the implementation process | - Make an implementation plan to adapt the national recommendations to local context - Define local stakeholders - Tailor the implementation strategies to the local determinants |
| Use evaluative and iterative strategies | Support in the implementation process    Knowledge on fall prevention and implementation | - Identify barriers and facilitators - Audit and provide feedback - Develop a formal implementation blueprint |
| Provide interactive assistance | Support in the implementation process | - Facilitation digital and face-to-face |
| Develop stakeholder interrelationships | Establishing a resource team from different professions and levels    Manager commitment | - Identify and prepare Champions - Organize clinician resource team meetings - Use an implementation advisor - Education on the role of leadership in implementation |
| Train and educate stakeholders | Knowledge on fall prevention and implementation | - Develop educational materials on fall prevention and implementation - Make training dynamic - Distribute educational materials on fall prevention and implementation - Create a learning collaborative - Conduct educational meetings for clinicians |
| Support clinicians | Knowledge on fall prevention and implementation    Management commitment    Support in the implementation process | - Remind clinicians - Conduct educational meetings for managers and health professionals - Make educational material available |
