# Supplementary material for: Tailored implementation of national recommendations on fall prevention among older adults in municipalities in Norway (FALLPREVENT trial): a study protocol for a cluster-randomised trial
Source: Implement Sci. 2024 Jan 25;19:5. doi: 10.1186/s13012-024-01334-2 (PMC10811923; doi:10.1186/s13012-024-01334-2)
Supplement: Supplementary file 2 — Additional file 2: Table S2. Developing a local implementation plan. [file 13012_2024_1334_MOESM2_ESM.docx]

**Table S2.** Developing a local implementation plan.

| **Tasks:** | **Description:** |
| --- | --- |
| Identify Problems/determine the know-do gap | 1. Evaluate the current practice and the recommendations for preventing falls by using the “Gap Analysis” tool.  2. Determine local aims for the implementation and decide how to monitor use and evaluate the success of implementation. |
| Adapt knowledge to local context | 1. Assess usefulness and appropriateness of the recommendations to the local settings and circumstances by use of a stakeholder analysis identifying influence and support of relevant stakeholders. |
| Identify local barriers and facilitators | 1. Identify local barriers and facilitator by use of a tool for identifying barriers and facilitators. |
| Select, tailor, and implement interventions to promote fall prevention | 2. Select, tailor, and implement interventions to promote fall prevention, where the tailoring strategies are built on the local barriers identified in the past step. Furthermore, the strategies identified from the co-creation process targeting leader involvement and educational enhancement will be adjusted to fit the needs of the municipalities/city district. |
